# Endoplasmic reticulum stress induces apoptosis of arginine vasopressin neurons in central diabetes insipidus via PI3K/Akt pathway

**DOI:** 10.1111/cns.13089

**Published:** 2019-01-24

**Authors:** Ming‐Feng Zhou, Zhan‐Peng Feng, Yi‐Chao Ou, Jun‐Jie Peng, Kai Li, Hao‐Dong Gong, Bing‐Hui Qiu, Ya‐Wei Liu, Yong‐Jia Wang, Song‐Tao Qi

**Affiliations:** ^1^ Department of Neurosurgery Nanfang Hospital, Southern Medical University Guangzhou China; ^2^ The First School of Clinical Medicine Southern Medical University Guangzhou China

**Keywords:** apoptosis, central diabetes insipidus, central nervous system, drug target, PI3K/Akt pathway

## Abstract

**Aims:**

Central diabetes insipidus (CDI), a typical complication caused by pituitary stalk injury, often occurs after surgery, trauma, or tumor compression around hypothalamic structures such as the pituitary stalk and optic chiasma. CDI is linked to decreased arginine vasopressin (AVP) neurons in the hypothalamic supraoptic nucleus and paraventricular nucleus, along with a deficit in circulating AVP and oxytocin. However, little has been elucidated about the changes in AVP neurons in CDI. Hence, our study was designed to understand the role of several pathophysiologic changes such as endoplasmic reticulum (ER) stress and apoptosis of AVP neurons in CDI.

**Methods:**

In a novel pituitary stalk electric lesion (PEL) model to mimic CDI, immunofluorescence and immunoblotting were used to understand the underlying regulatory mechanisms.

**Results:**

We reported that in CDI condition, generated by PEL, ER stress induced apoptosis of AVP neurons via activation of the PI3K/Akt and ERK pathways. Furthermore, application of N‐acetylcysteine protected hypothalamic AVP neurons from ER stress‐induced apoptosis through blocking the PI3K/Akt and ERK pathways.

**Conclusion:**

Our findings showed that AVP neurons underwent apoptosis induced by ER stress, and ER stress might play a vital role in CDI condition through the PI3K/Akt and ERK pathways.

## 1 INTRODUCTION

The hypothalamo‐neurohypophyseal system (HNS), especially the pituitary stalk, serves as an essential role in water‐electrolyte regulation. Injury of the pituitary stalk could lead to imbalance of HNS and even cause severe hypothalamic dysfunction.^1^, ^2^ Among these, central diabetes insipidus (CDI), characterized by polyuria, polydipsia, and low urine specific gravity, is a typical complication after surgery, trauma, or tumor around the hypothalamus.^3^, ^4^

In this work, we used a pituitary stalk electrical lesion (PEL) model to induce CDI condition, by targeting pituitary stalk without interrupting the adenohypophysis and portal circulation based on previous studies.^3^, ^5^, ^6^ Several former studies have reported a decreased number of arginine vasopressin (AVP) neurons in the hypothalamic nuclei and a production of apoptotic AVP neurons after hypophysectomy, but the underlying mechanism remains elusive.^6^, ^7^ Meanwhile, numerous studies have verified the effect of endoplasmic reticulum (ER) stress on inducing cell apoptosis in other issues.^9^, ^10^ In order to illustrate that the ER stress was involved in the apoptosis of AVP neurons, we examined the changes in magnocellular AVP neurons in the hypothalamic‐pituitary axis, including the supraoptic nucleus (SON) and paraventricular nucleus (PVN) after PEL.

Furthermore, considering that the mechanism underlying the apoptosis of AVP neurons remains unclear, we also investigated several apoptosis‐associated pathways, intending to identify the specific regulatory pathways that could serve as potential therapeutic targets as well.

## 2 MATERIALS AND METHODS

### 2.1 Animals

Male Sprague‐Dawley rats with an average body weight of 200 g (180‐220 g) were housed in independent metabolic cages with a daily light and dark cycle in a temperature‐controlled room. During the whole experiment, food and water were provided without restriction. All procedures were in accordance with our institutional guidelines and were approved by the regional ethics committee in advance.

### 2.2 Pituitary stalk electric lesion surgery

All animals were housed in independent metabolic cages for 3 days to accommodate environment before surgery. PEL surgery was performed according to our previous studies.^7^, ^8^ In brief, rats were first anesthetized with 5% isoflurane with the gas mask fitted into the platform of the stereotaxic instrument (Stoelting). Anesthesia was maintained with 1.5%‐2% isoflurane delivered in air at 0.5 L/min during the whole surgery process. Then, rats were mounted on a stereotaxic frame with nose down 3.3 mm and the skull was opened by removing a 3 by 3 mm square (approx.) of bone with center at 3.8‐mm caudal to bregma in the sagittal midline. Next, a 3D‐printed lesion knife, with a 2.5‐mm‐wide curved head and 1‐mm‐thickness, was lowered in the sagittal midline until it reached the floor of the skull base which was over 8 mm beneath the surface of the brain. To perform PEL, a cathodic current of 500 µA was applied for 40 seconds with a constant power supply output apparatus (53500, UGO Basile, Italy), while no current was applied in sham surgery. After surgery, all rats were put back to metabolic cages and the daily water consumption, daily urine volume, and urine specific gravity were monitored for 14 days.

### 2.3 Administration of N‐acetylcysteine

N‐acetylcysteine (NAC) (Sigma, USA) was dissolved in normal saline to get final concentration of 100 mg/mL. In the experiment of NAC treatment, rats were divided into 2 groups randomly: PEL+ saline, and PEL+ NAC. In NAC treatment group, all rats received NAC (200 mg/kg body weight) while saline treatment rats received saline instead, for continuous 3 or 7 days after surgery based on the experimental design.

### 2.4 Perfusion and tissue processing for immunohistochemistry

At the end of the postoperative experimental period, the rats were deeply anesthetized with sodium pentobarbital (80 mg/kg), then were perfused intracardially with precool normal saline, followed by cool 4% paraformaldehyde in phosphate buffer saline (PBS; pH 7.4). For coronal cryostat brain sectioning, brains were removed from the skull and placed in 4% formaldehyde for at least 24 hours. After fixation, brains were transferred to sequential 15% and 30% sucrose at 4°C for at least 24 hours, respectively. Then, brains were embedded in OCT compound, and coronal sections were cut at 40 µm on a freezing microtome. All sections were collected and rinsed in PBS.

### 2.5 Immunofluorescence

For coronal frozen brain section immunofluorescence staining, sections were rinsed with PBS, followed by blocked with 5% nonspecific antigen goat serum for 1 hour at 37°C. Then, the sections were incubated overnight at 4°C with specific primary antibodies. In the next day, after rinsed with PBS containing 0.5% Triton X‐100 for three times, sections were incubated for 1 hour at 37°C with corresponding secondary antibodies conjugated with Alexa 488 or Alexa 594 (Thermo Fisher Scientific, USA). The primary and secondary antibodies were diluted in PBS containing 5% normal goat serum and 0.2% Triton X‐100. After incubation with secondary antibodies, the sections were mounted on glass slides and cover glasses were slipped in mounting medium. Fluorescent images were captured with a confocal microscope (LSM880, Zeiss, Germany). For cell number counting, the data were presented as the number of cells/mm^2^. For double staining analysis, the data were presented as the ratio of number of double‐positive cells to number of single‐positive cells.

### 2.6 Western blot

Western blot was performed using hypothalamic tissue samples. Briefly, at the end of the postoperative experimental period, the rats were deeply anesthetized with sodium pentobarbital (80 mg/kg) and then were perfused intracardially with precool normal saline. Next, brains were removed from the skull and hypothalamus nuclei including SON and PVN were observed under a microscope and separated by forceps. Then, tissues were washed thoroughly with PBS for three times and lysed with RIPA buffer (50 mmol/L Tris‐HCl pH 8.0, 1 mmol/L EDTA pH 8.0, 5 mmol/L DTT, 2% SDS) containing protease inhibitor and phosphoric acid protease inhibitor at 4°C for 30 minutes. The protein concentration was determined using BCA assay kit (Beyotime Inc, China). Protein samples were separated subsequently using SDS‐PAGE gel and electro‐transferred to polyvinylidene difluoride (PVDF) membranes (Millipore, USA). Then, the membranes were blocked with 5% bovine serum albumin and then incubated with primary antibodies overnight at 4°C. In the next day, after washed with TBST, membranes were reacted with corresponding horseradish peroxidase‐conjugated secondary antibodies for 1 hour under room temperature. Finally, signals were detected using enhanced chemiluminescence reagents and images were captured with a digital camera (Pierce, Rockford, IL, USA).

### 2.7 Antibodies

The following primary antibodies were used: rabbit anti‐AVP (AB1565; Millipore); mouse anti‐AVP (MABN845; Millipore); mouse anti‐CHOP (sc‐7351; Santa Cruz, USA); rabbit anti‐PI3K (41339; SAB, USA); rabbit anti‐p‐PI3K (phosopho‐Tyr467/199) (11508; SAB); rabbit anti‐Akt (33748; SAB); rabbit anti‐p‐Akt (phospho‐Ser473) (11054; SAB); mouse anti‐ERK1/2 (AF1051; Beyotime); mouse anti‐p‐ERK1/2 (phosopho‐Thr202/Tyr204) (AF1891; Beyotime); rabbit anti‐p‐JNK (phospho‐Thr183/Tyr185) (4668, CST, USA); mouse anti‐BAD (AB008; Beyotime); rabbit anti‐Caspase3 (19677‐1‐AP; Proteintech, USA); rabbit anti‐PERK (20582‐1‐AP; Proteintech)； rabbit anti‐XBP‐1 (25997‐1‐AP; Proteintech)； rabbit anti‐ATF‐6 (24169‐1‐AP; Proteintech)； rabbit anti‐ATF‐4 (10835‐1‐AP; Proteintech)； rabbit anti‐eIF2α (11233‐1‐AP; Proteintech); β‐actin (CW0264; CWbio, China); and β‐tubulin (CW0098; CWbio).

### 2.8 Statistical analysis

The data between groups were compared by one‐way ANOVA combined with LSD using SPSS 23.0 (IBM, USA) and GraphPad 7.0 software. Two‐tailed Student's test was used for single comparison between two groups. *P* values for multiple comparisons were adjusted using LSD correction. Error bars in all figures were presented as the mean ± SEM. *P* < 0.05 was considered significant.

## 3 RESULTS

### 3.1 Typical tri‐phasic central diabetes insipidus after PEL surgery

**Figure 1:**
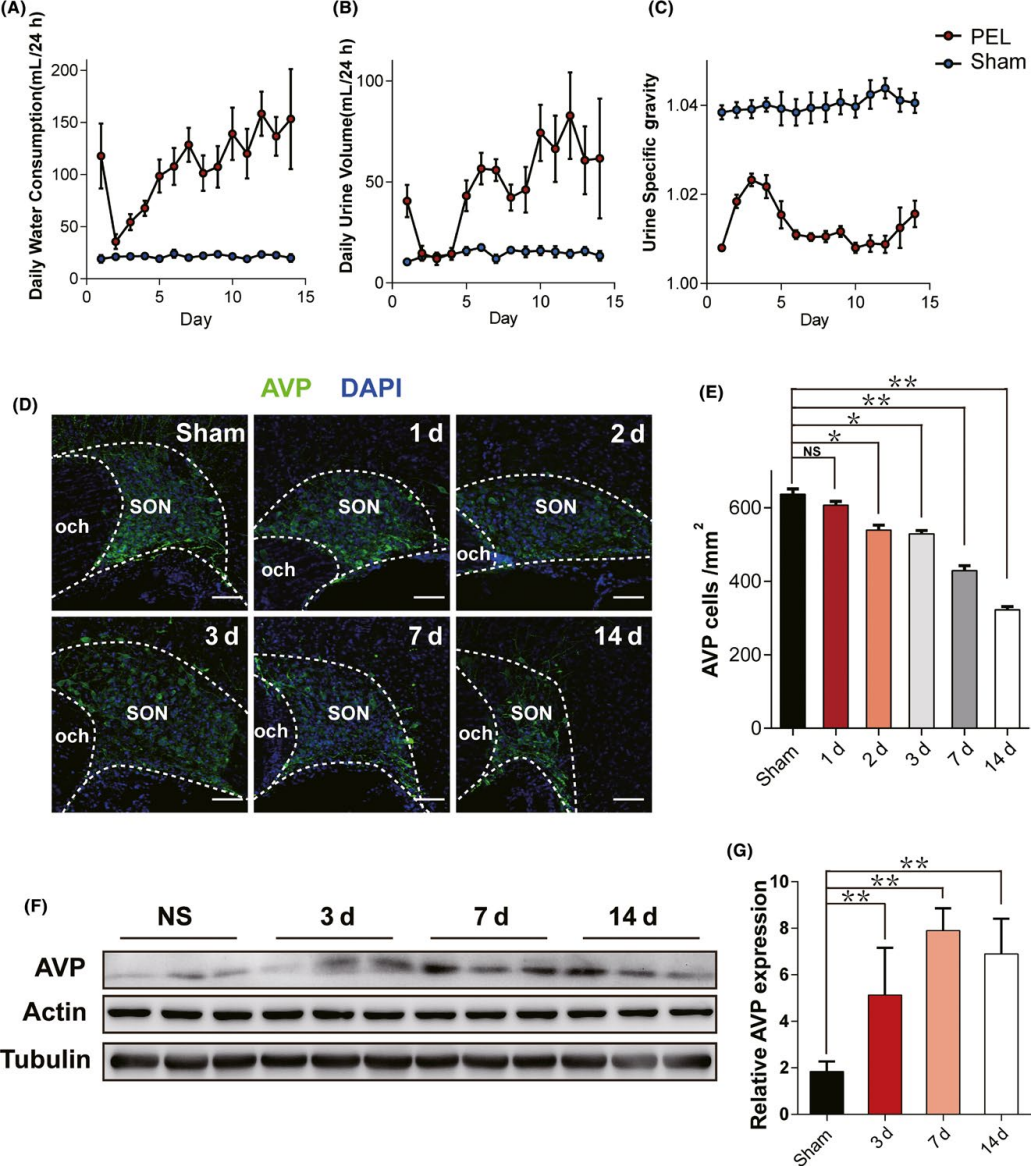
Characteristics of biological parameters after PEL surgery. A, Daily water consumption (DWC), (B) daily urine volume (DUV), and (C) urine specific gravity (USG) during 14 days after surgery in PEL surgery rats (N = 7) and sham‐operated rats (N = 6). D, Time course immunofluorescent patterns of residual AVP neurons at day 1 (N = 3), day 2 (N = 3), day 3 (N = 3), day 7 (N = 3), and day 14(N = 3) postsurgery in PEL and sham‐operated rats (N = 6), respectively. E, Quantification of D; **P < 0.01 compared to sham‐operated rats, *P < 0.05 compared to sham‐operated rats; scale bar, 100 μm. F, Time course expression of AVP in hypothalamic tissue samples at day 3 (N = 3), day 7 (N = 3), and day 14 (N = 3) postsurgery in PEL and sham‐operated rats (N = 3), respectively. Actin and tubulin were used as loading controls. G, Quantification of F; **P < 0.01 compared to sham‐operated rats. AVP, arginine vasopressin; SON, supraoptic nucleus; PVN, paraventricular nucleus

To evaluate the severity of CDI, we collected three major biologic parameters: daily water consumption (DWC), daily urine volume (DUV), and urine specific gravity (USG) continuously for 14 days after PEL surgery. As shown in Figure 1A‐C, during whole experiment period, sham‐operated rats showed a relatively stable condition with a DWC of 22.7 ± 2.0 mL/24 h, a DUV of 15.8 ± 2.0 mL/24 h, and a USG of 1.044 ± 0.002, respectively. However, the rats that underwent PEL surgery exhibited a typical tri‐phasic pattern based on DWC, DUV, and USG. On the first day after PEL surgery, rats showed an extreme increase in DWC (117.9 ± 31.3 mL/24 h) and DUV (40.6 ± 8.0 mL/24 h) with a sharp decrease in USG (1.008 ± 0.006). From the day 2 to day 4 postsurgery, DWC and DUV declined remarkably to 35.7 ± 7.2 mL/24 h and 14.7 ± 3.7 mL/24 h, while USG increased to 1.018 ± 0.001 simultaneously. Next, during days 5‐14 postsurgery, DWC and DUV increased again, peaked at day 10 postsurgery and then reached a relatively stable condition with a DWC of 120.0 ± 23.8 mL/24 h, a DUV of 66.4 ± 16.5 mL/24 h, and a USG of 1.009 ± 0.002.

Next, in order to investigate the underlying mechanism of the typical tri‐phasic pattern of CDI, we counted the number of AVP neurons in SON at sequential time points after PEL surgery. The results showed a significant decrease in number of AVP neurons at day 7 (427.2 ± 5.3/mm^2^) and day 14 (312.1 ± 4.6/mm^2^) postsurgery compared to sham‐operated rats (625.2 ± 16.7/mm^2^; Figure 1D,E) with a same phenomenon found in PVN (data not shown). Moreover, AVP expression level in hypothalamus tissue was found significantly upregulated in PEL‐operated rats, which might be a compensatory response to the downstream pituitary stalk injury. Interestingly, AVP expression level was found up‐regulated at day 3, similar to day 7 and day 14 postsurgery (Figure 1F,G), which we concluded to be an acute stress to an AVP neuronal fiber injury.

### 3.2 ER stress was involved in a time course pattern of hypothalamic AVP neuron apoptosis in SON and PVN

**Figure 2:**
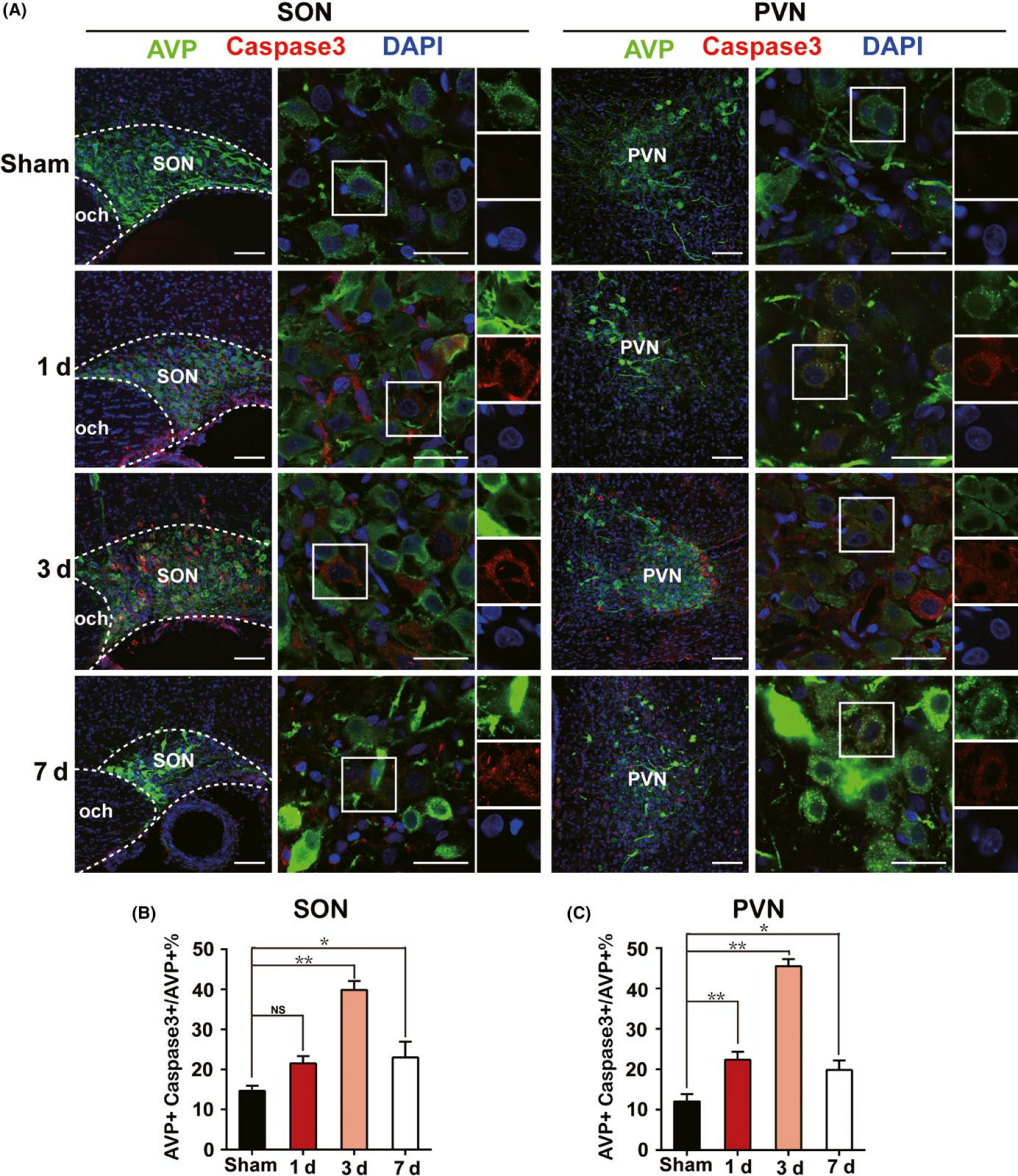
Emerging of apoptotic AVP neurons in acute phase after PEL surgery. A, Immunofluorescent analysis of apoptotic AVP neurons of SON or PVN characterized by AVP+ Caspase3+ neurons at day 1 (N = 3), day 3 (N = 3), and day 7 (N = 3) after PEL surgery compared with sham‐operated rats (N = 3), respectively. B, Quantification of SON in A. C, Quantification of PVN in A; **P < 0.01 compared to sham‐operated rats, *P < 0.05 compared to sham‐operated rats; scale bars, 100 μm for low magnification image, 30 μm for high magnification image. AVP (green), Caspase3 (Red), DAPI (blue). AVP, arginine vasopressin; SON, supraoptic nucleus; PVN, paraventricular nucleus

A previous study has demonstrated that apoptosis was involved in AVP neurons change after hypophysectomy.^6^ Therefore, we examined the apoptosis pattern of AVP neurons both in SON and PVN after PEL surgery as well. As shown in Figure 2, immunofluorescent imaging showed a high number of Caspase3+ AVP neurons immediately after PEL surgery. Cell number of Caspase3+ AVP neurons peaked at day 3 (39.9% ± 2.2% in SON and 45.6% ± 1.7% in PVN), declined to 23.0% ± 3.9% in SON and 19.9% ± 2.3% in PVN at day 7, compared to 14.7% ± 1.3% in SON and 12.1% ± 1.8% in PVN in sham‐operated rats.

**Figure 3:**
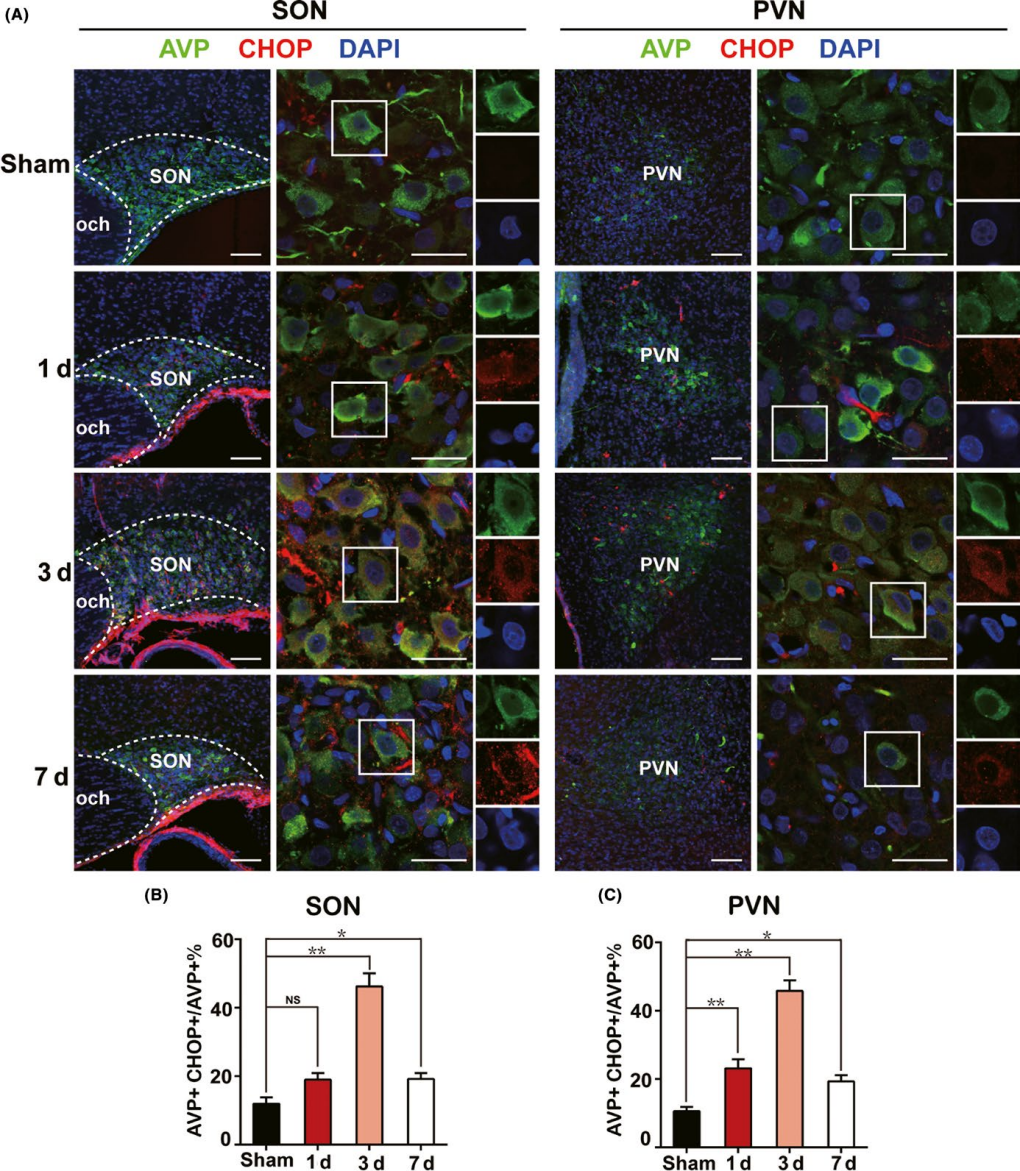
Triggered ER stress in AVP neurons in acute phase after PEL surgery. A, Immunofluorescent images visualized that ER stress was triggered in AVP neurons of SON or PVN characterized by AVP+ CHOP+ neurons at day 1 (N = 3), day 3 (N = 3), and day 7 (N = 3) postsurgery in PEL and sham‐operated rats (N = 3), respectively. B, Quantification of SON in A. C, Quantification of PVN in A; **P < 0.01 compared to sham‐operated rats, *P < 0.05 compared to sham‐operated rats; scale bars, 100 μm for low magnification image, 30 μm for high magnification image. AVP (green), CHOP (Red), DAPI (blue). AVP, arginine vasopressin; SON, supraoptic nucleus; PVN, paraventricular nucleus

**Figure 4:**
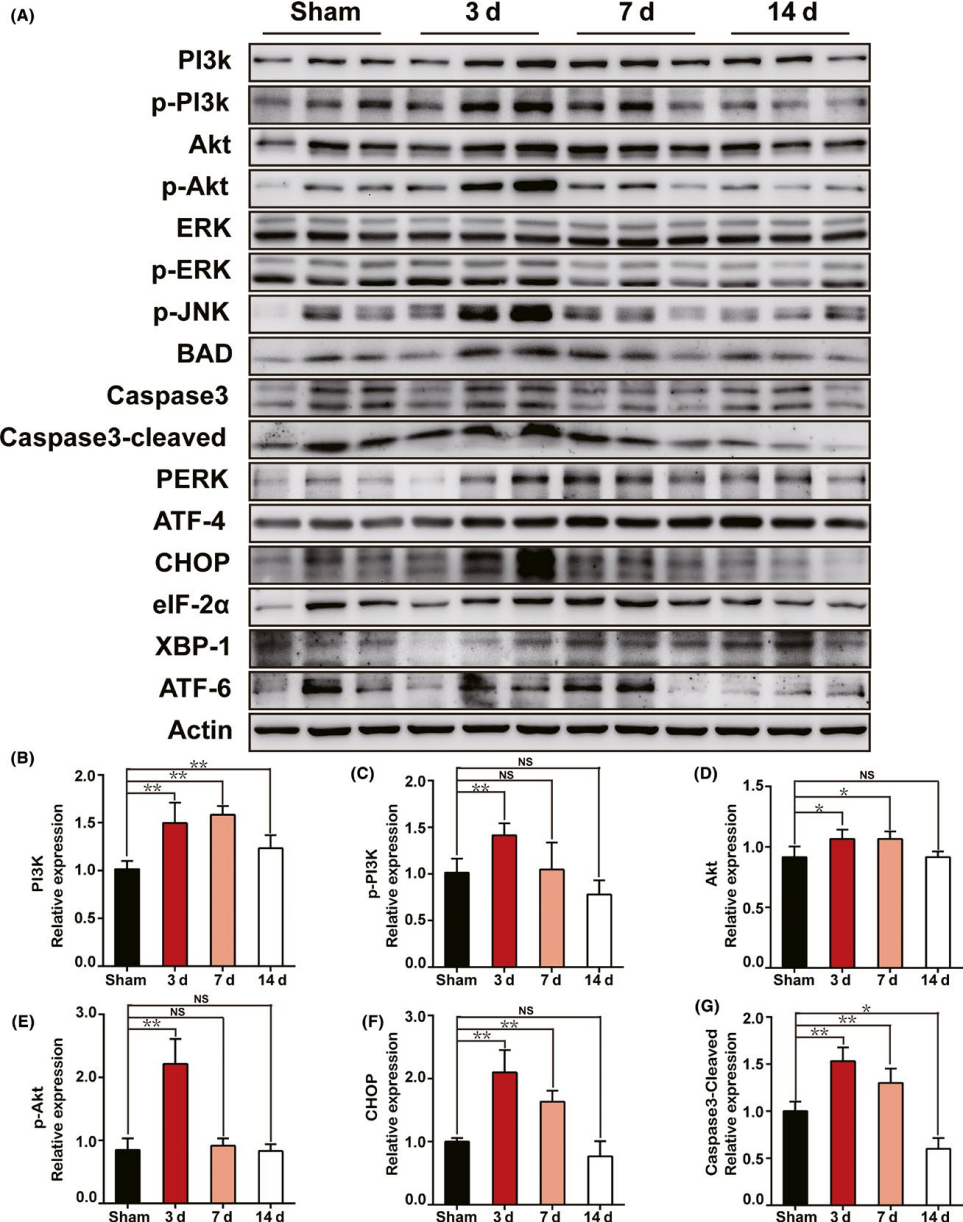
Time course activation of the PI3K/Akt signaling pathway could trigger ER stress‐induced apoptosis in SON and PVN after PEL surgery. A, Protein levels of PI3K/Akt, ERK, ER stress, and apoptosis signaling pathway markers were measured at day 3 (N = 3), day 7 (N = 3), and day 14 (N = 3) postsurgery in PEL and sham‐operated rats (N = 3) by Western blot, respectively. Actin was used as a loading control. B‐G, Quantification of A; **P < 0.01 compared to sham‐operated rats, *P < 0.05 compared to sham‐operated rats

Endoplasmic reticulum stress was reported by several studies to be associated with apoptosis in many issues^11^, ^12^ through different mechanisms including calcium overload,^16^, ^17^ reactive oxygen toxicity,^18^, ^19^ and so on. As shown in Figure 3, we unexpectedly found that C/EBP homologous protein (CHOP), an essential marker of ER stress, presented a similar expression pattern to Caspase3, with a portion of CHOP+ AVP neurons of 19.0% ± 1.9% in SON and 23.2% ± 2.7% in PVN at day 1, 46.3% ± 2.8% in SON and 45.9% ± 3.0% in PVN at day 3, and 19.2% ± 1.8% in SON and 19.3% ± 1.8% in PVN at day 7 after PEL surgery, respectively, compared to 12.0% ± 1.8% in SON and 10.6% ± 1.2% in PVN in sham‐operated rats. Moreover, we examined three canonical ER stress signaling pathways, including PERK‐eIF2α‐ATF4‐CHOP, IRE1‐XBP1, and ATF6‐ERAD. The results showed a significant increase in PERK, eIF2α, ATF4, and CHOP expression especially at day 3 postsurgery, which indicated a major role of PERK‐eIF2α‐ATF4‐CHOP signaling pathway in ER stress regulation after PEL surgery. However, IRE1‐XBP1 and ATF6‐ERAD showed less activation, characterized by the moderate upregulation of XBP1 and ATF6 (Figure 4). Further, immunoblotting results also showed high expression of Caspase3/cleaved‐Caspase3 and BAD, further supporting the immunofluorescence results and confirming the apoptotic pattern of AVP neurons (Figure 4A,G). Taken together, our data indicated that ER stress in the early stage partly contributed to the apoptosis of AVP neurons after PEL surgery.

### 3.3 PI3K/Akt and ERK pathways might mediate the ER stress and apoptosis after PEL

To investigate the underlying mechanisms of ER stress and apoptosis after PEL surgery, we collected protein samples from SON and PVN tissues to examine several classical apoptosis and ER stress‐associated signaling pathways, including PI3K/Akt and ERK pathways. As shown in Figure 4A‐E, PI3K, p‐PI3K, Akt, and p‐Akt showed an increase after PEL surgery over the time course. PEL‐induced activation of the PI3K/Akt pathway demonstrated high expression of p‐PI3K and p‐Akt, peaked at day 3‐7, and gradually decreased to baseline at day 14 after PEL surgery. We also measured the expression of ERK1/2, which has been previously reported to be linked to neuronal apoptosis.^20^ We found that PEL surgery also led to activation of the ERK pathway, characterized by a moderate increase in ERK and p‐ERK expression at day 3 followed by a gradual decrease at day 7 and day 14 after PEL surgery. Furthermore, we detected the expression of several essential apoptosis‐associated proteins, such as Caspase3, cleaved‐Caspase3, p‐JNK, and BAD, to verify the apoptosis after PEL. All apoptosis‐associated proteins showed a similar time course expression pattern, with a sharp increase in acute phase after PEL surgery, a peak at day 3 and a gradual decline at day 7 and day 14, in agreement with that found in PI3K/Akt and ERK pathways, compared to sham‐operated rats (Figure 4A,G). In summary, our data suggested that PEL might induce ER stress and apoptosis of AVP neurons via PI3K/Akt and ERK pathways.

### 3.4 Effect of NAC on the ER stress and apoptosis of SON and PVN through PI3K/Akt and ERK pathways during acute phase after PEL

**Figure 5:**
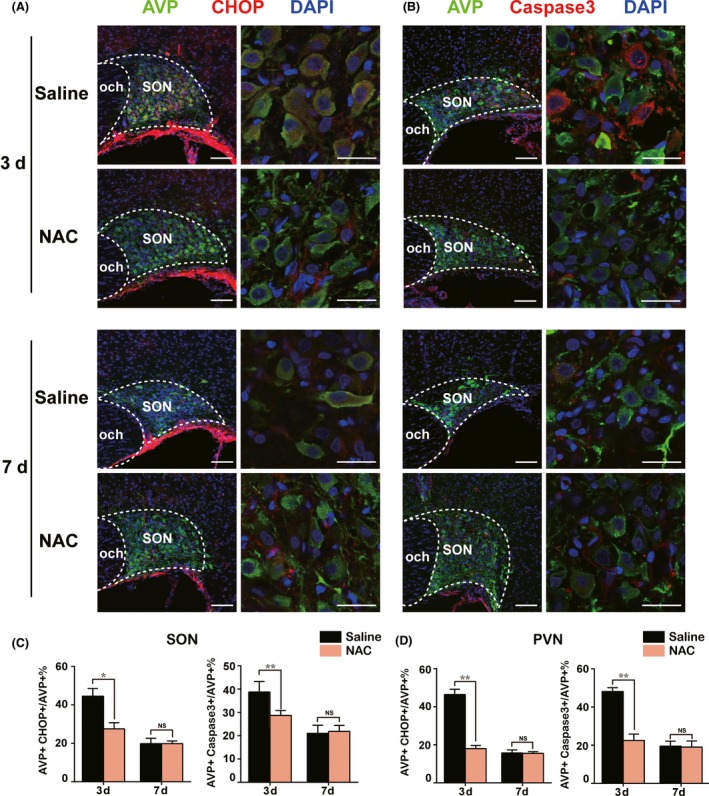
N‐acetyl‐cysteine (NAC) alleviated activation of ER stress and protected AVP neurons from apoptosis after PEL surgery in hypothalamus. A, NAC significantly alleviated activation of ER stress in AVP neurons at day 3, while no difference was found at day 7 after PEL surgery, as confirmed by immunofluorescent analysis of CHOP+ AVP+ neurons of SON, compared to saline‐treated PEL rats. B, NAC significantly attenuated generation of apoptotic AVP neurons at day 3 while no difference was found at day 7 after PEL surgery and NAC treatment, as confirmed by immunofluorescent analysis of AVP+ Caspase3+ apoptotic AVP neurons of SON, compared with saline‐treated PEL rats. C, Quantification of SON in A and B. D, Quantification results of the effects of NAC on ER stress and apoptosis of PVN (immunofluorescent images not shown). **P < 0.01 compared to saline‐treated PEL rats, *P < 0.05 compared to saline‐treated PEL rats; scale bars, 100 μm for low magnification image, 30 μm for high magnification image. AVP (green), CHOP (Red), DAPI (blue) in A. AVP (green), Caspase3 (Red), DAPI (blue) in B. AVP, arginine vasopressin; SON, supraoptic nucleus; PVN, paraventricular nucleus

**Figure 6:**
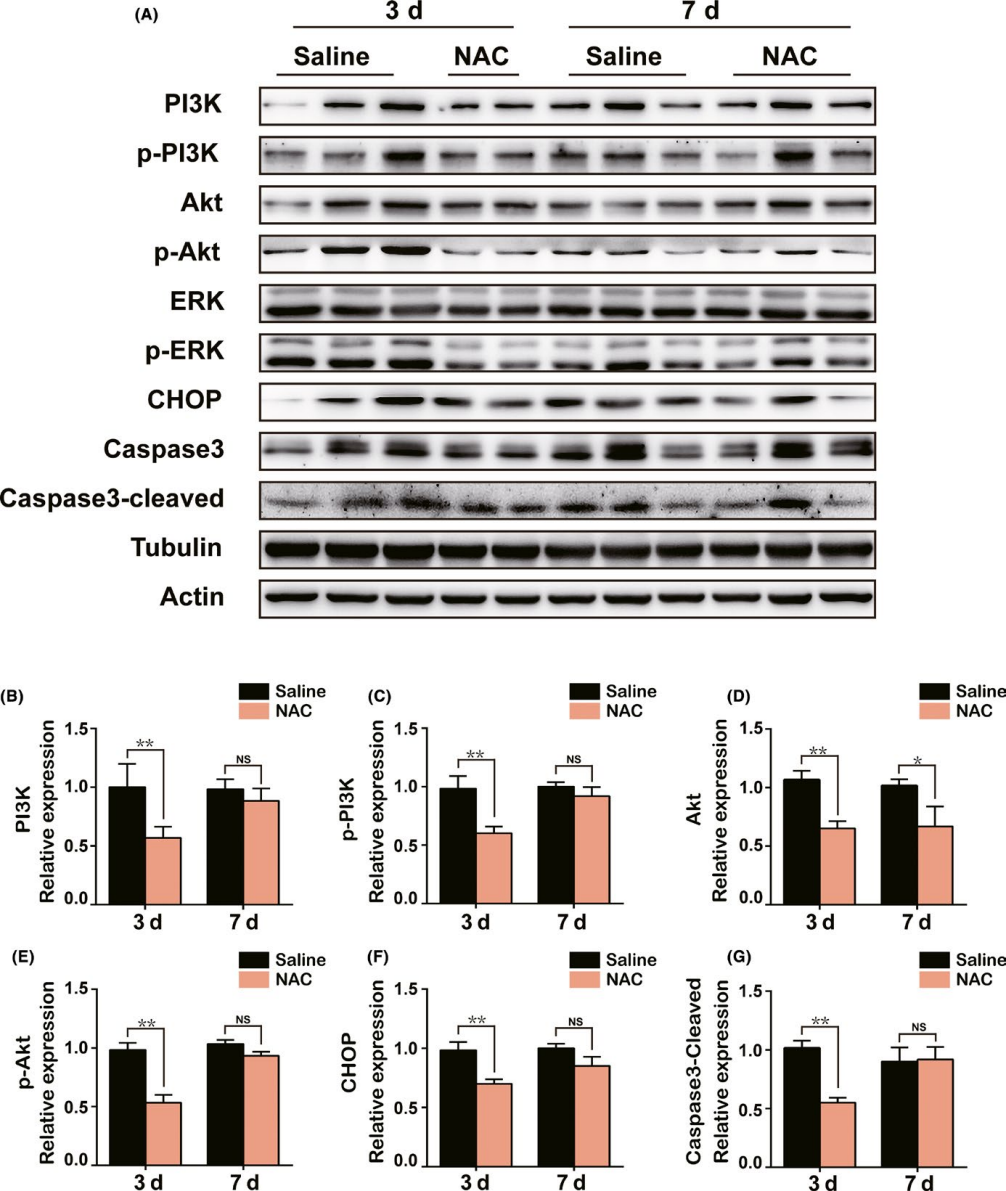
Early NAC treatment could rescue AVP neurons from ER stress‐induced apoptosis through inhibition of the PI3K/Akt and ERK signaling pathways. A, Protein levels of PI3K/Akt, ERK, ER stress, and apoptosis signaling pathway markers were measured in hypothalamus tissue samples from saline (3 days) +PEL (N = 3), NAC (3 days) +PEL (N = 2), saline (7 days) +PEL (N = 3), and NAC (7 days) +PEL (N = 3) by Western blot, respectively. Actin and tubulin were used as loading controls. B‐G, Quantification of A; **P < 0.01 compared to saline‐treated PEL rats, *P < 0.05 compared to saline‐treated PEL rats

The above data suggested that ER stress may contribute to the PEL‐induced apoptotic effect on hypothalamic AVP neurons. To further support our hypothesis, we tested whether treatment of rats with drug targeting ER stress could rescue hypothalamic AVP neurons from apoptosis after PEL. We intraperitoneally injected rats with NAC, an antioxidant of ER stress, for three or seven continuous days immediately after PEL surgery. As shown in Figure 5, immunofluorescent images showed that NAC treatment significantly abolished the activation of ER stress, characterized by a low ratio of CHOP+ AVP neurons in NAC‐injection rats (31.0% ± 3.7% in SON, 18.0% ± 1.6% in PVN) compared to 44.5% ± 4.0% in SON and 46.3% ± 2.8% in PVN in the saline‐injection rats at day 3 after PEL surgery. Furthermore, we determined the apoptosis pattern of AVP neurons in SON and PVN after administration of NAC. Apoptosis ratio of AVP neurons was 38.3% ± 1.0% in SON and 48.1% ± 2.0% in PVN in saline‐injection rats, while the ratio declined to 29.5% ± 1.0% in SON and 22.5% ± 3.4% in PVN in NAC‐injection rats at day 3 after PEL surgery. However, no difference was detected between saline‐injection rats and NAC‐injection rats in the ER stress and apoptosis pattern at day 7 after PEL surgery. In regard to the mechanism, we asked whether NAC treatment could rescue AVP neurons from the ER stress‐induced apoptosis via inhibition of the PI3K/Akt and ERK pathways after PEL surgery. Immunoblotting results verified that NAC treatment could prevent the activation of the PI3K/Akt and ERK1/2 pathways, characterized by decreased expression of PI3K, p‐PI3K, Akt, p‐Akt, ERK, and p‐ERK (Figure 6A‐E). Furthermore, low expression of Caspase3, cleaved‐Caspase3, and CHOP based on immunoblotting results further validated the protective effect of NAC in the acute phase after PEL surgery (Figure 6A,F,G). Taken together, our data provided evidence that NAC treatment could inhibit the ER stress via blocking the activation of the PI3K/Akt and ERK pathways in the acute phase and rescued AVP neurons from apoptosis after PEL surgery.

## 4 DISCUSSION

In our study, we revealed that ER stress triggered by activation of the PI3K/Akt and ERK pathways activation could lead to apoptosis of AVP neurons in CDI generated by PEL surgery. Furthermore, NAC treatment could inhibit the PI3K/Akt and ERK pathways and protect hypothalamic AVP neurons from ER stress‐induced apoptosis, which could serve as a new potential therapeutic target of CDI and hypothalamus injury.

### 4.1 PEL surgery constructed a suitable model for hypothalamic‐hypophyseal system study

Balanced arginine vasopressin release relies on an intact HNS, as arginine vasopressin is synthesized in several hypothalamic nuclei including SON and PVN, then transported to neurohypophysis for storage through neuronal axons near median eminence, and finally released into circulation by hypophyseal portal system.^21^, ^22^ Any injury of HNS could lead to dysfunction of the whole body and even result in severe diseases. Hypothalamic injury caused by trauma, tumor, or surgery around pituitary stalk serves as the common reasons of imbalance of HNS.^24^, ^25^

Central diabetes insipidus usually occurs as a result of the disturbance of the HNS, especially when pituitary stalk is implicated during tumor resection surgery or other operations near the hypothalamus.^21^ Typically, CDI can be separated into three distinct stages: the acute polyuria stage, the oliguria stage, and the recovery stage.^26^, ^27^ The polyuria stage, usually occurring immediately after injury, is characterized by an extremely high intake of water along with an extremely high urine output and an extremely low urine specific gravity. The oliguria stage, following the polyuria stage, is featured by a sharp drop of water intake and urine output. The recovery stage is the long‐term period with water intake, urine output, and urine specific gravity gradually returning to the baseline.

In the present study, we used a PEL model targeting the pituitary stalk through the transparietal approach to mimic CDI, without interfering with the anterior pituitary lobe and hypophyseal portal system. Rats showed a typical tri‐phasic pattern CDI after PEL surgery, featured by the polyuria stage at day 1 postsurgery, the oliguria stage from day 2 to day 4 and the recovery stage from day 5 after PEL surgery. Our study not only demonstrated that PEL provided a relevant animal model to study CDI but also provided a new approach to study axon injury as well as axon repair or regeneration, because the pituitary stalk also served as the axon of upstream hypothalamic nuclei including SON and PVN.

### 4.2 Involvement of ER stress in neuronal apoptosis

Endoplasmic reticulum stress, triggered as a result of homeostasis disequilibrium, could lead to accumulation of misfolded proteins in the endoplasmic reticulum lumen and initiate cell apoptosis in many issues.^10^, ^11^, ^13^, ^29^ Despite overwhelming evidence reporting that ER stress could serve as a critical component of the pro‐apoptotic signaling pathway in the central nervous system injury^30^, ^31^ and several studies identifying the role of ER stress in myelinated nerve fibers during trauma or neurodegeneration disease,^33^ little research has been carried out to elucidate the effect of ER stress‐induced apoptosis in unmyelinated nerve fibers, especially secretory neurons or secretory nerve fiber. Here, in our PEL‐induced CDI model, we demonstrated that in secretory AVP neurons, ER stress may also initiate apoptosis, in agreement with evidence found in other issues. ER stress was reported to be regulated by three canonical signaling pathways: PERK‐eIF2α‐ATF4‐CHOP, IRE1‐XBP1, and ATF6‐ERAD, in various issues.^34^ Further, several studies have reported that the PERK‐eIF2α‐ATF4‐CHOP signaling pathway plays an essential role in ER stress‐induced apoptosis, especially in neuronal issues including spinal cord injury, subarachnoid hemorrhage injury, and cerebral ischemia injury.^35^, ^36^ In the present study, our data indicated consistent involvement of PERK‐eIF2α‐ATF4‐CHOP in ER stress‐induced apoptosis in CDI generated by PEL surgery.

Although ER stress‐induced apoptosis has been confirmed by many researchers, the underlying mechanism still remains unclear.^19^, ^32^ To identify the potential pathways mediating ER stress‐induced AVP neuronal apoptosis, we investigated several classical apoptosis and ER stress‐associated signaling pathways, including the PI3K/Akt and ERK pathways. Interestingly, we found that the PI3K/Akt pathway showed similarities with ER stress and apoptosis patterns due to increased activation in the acute phase but relatively decreased activation over the long‐term in CDI rats.

Taken together, our results showed that activation of the PI3K/Akt and ERK pathways may trigger ER stress, exacerbate cell vulnerability to injury, and finally promote AVP neuron apoptosis in CDI.

### 4.3 Therapeutic target for ER stress and apoptosis in CDI

N‐acetylcysteine, a classical antioxidant drug used in chronic obstructive pulmonary disease and contrast‐induced nephropathy, was involved in the metabolism of glutamate, cysteine, and glutathione.^40^ NAC was reported to protect cells from oxidative stress, inflammation, and apoptosis in many issues including neuronal cells.^40^, ^41^ In the present study, we demonstrated that NAC treatment could also prevent activation of ER stress via blocking the PI3K/Akt and ERK pathways and consistently ameliorate apoptosis of AVP neurons in acute phase after PEL surgery. However, at the late stage of CDI, NAC treatment did not make a difference in deactivation of ER stress or apoptosis pattern. This may be due to the successful reversion of apoptosis by NAC in the early stage and that remodeling of residual cells rather than rescue of the injured cells should occur in the late stage of injury, in accordance with the biological rationale. Hence, our results indicated that early drug intervention may be beneficial to protect AVP neurons, highlighting the importance of early treatment intervention after neuronal injury. Taken together, our results suggested that the priority should be given to early protection of AVP neurons from ER stress‐induced apoptosis after PEL surgery, either by blocking the PI3K/Akt pathway or other potential therapeutic targets, which could be applied in other aspects of neuronal injury as well.

However, given the complexity of ER stress and apoptosis regulation, we only carried out a preliminary study to investigate its effect on CDI. Whether other mechanisms, including oxidative stress, calcium overload, and mitochondria disorder, were involved in ER stress and apoptosis regulation in CDI needs further investigation.

## 5 CONCLUSION

In our study, we demonstrated that triggering ER stress may lead to AVP neuron apoptosis via the PI3K/Akt and ERK pathways in the acute phase after CDI or hypothalamic injury. Importantly, we found that early NAC treatment rescued AVP neurons from ER stress‐induced apoptosis, thus providing a potential therapeutic target for CDI or central nervous system injury.

## CONFLICT OF INTEREST

All the authors declare that there was no conflict of interest.
